# An Interdigital Electrode Probe for Detection, Localization and Evaluation of Surface Notch-Type Damage in Metals

**DOI:** 10.3390/s18020371

**Published:** 2018-01-27

**Authors:** Lanshuo Li, Xiaoqing Yang, Yang Yin, Jianping Yuan, Xu Li, Lixin Li, Kama Huang

**Affiliations:** 1 School of Electronics and Information Engineering, Sichuan University, Chengdu 610065, China; lls@stu.scu.edu.cn (L.L.); yangyin@stu.scu.edu.cn (Y.Y.); kmhuang@scu.edu.cn (K.H.); 2 National Key Laboratory of Aerospace Flight Dynamics, Northwestern Polytechnical University, Xi’an 710129, China; jyuan@nwpu.edu.cn; 3 School of Electronics and Information, Northwestern Polytechnical University, Xi’an 710129, China; nwpu_lixu@126.com (X.L.); lilixin@nwpu.edu.cn (L.L.)

**Keywords:** interdigital electrode (IDE), metallic materials, microwave near-field detection, surface notch-type damage

## Abstract

Available microwave notch-type damage detection sensors are typically based on monitoring frequency shift or magnitude changes. However, frequency shift testing needs sweep-frequency data that make scanning detection becomes difficult and time-consuming. This work presents a microwave near-field nondestructive testing sensor for detecting sub-millimeter notch-type damage detection in metallic surfaces. The sensor is loaded with an interdigital electrode element in an open-ended coaxial. It is simple to fabricate and inexpensive, as it is etched on the RC4003 patch by using printed circuit board technology. The detection is achieved by monitoring changes in reflection amplitude, which is caused by perturbing the electromagnetic field around the interdigital structure. The proposed sensor was tested on a metallic plate with different defects, and the experimental results indicated that the interdigital electrode probe can determine the orientation, localization and dimension of surface notch-type damage.

## 1. Introduction

Nondestructive testing and evaluation (NDT&E) for defects in metal surfaces are especially crucial to those working in conditions that require high safety, such as ships and aircraft fuselages. Upon prolonged exposure to air and external impacts, there is a higher probability of causing destruction of these structures. Therefore, regular NDT&E for their safety is necessary. Several techniques have been developed to detect defects, such as acoustics [1,2], eddy-currents [3,4], and the magnetic method [5]. However, there are restrictions in practice. For instance, it is ineffective to detect defects hidden under coatings or under paint by these methods. While a class of technique based on microwave and millimeter wave developed rapidly, they have some irreplaceable superiority such as noncontact, non-pollution and the ability to penetrate non-metallic media [6,7,8], compared with other methods. 

However, some limitations still exist in microwave NDT&E systems. For instance, high cost [9], complication [10] and long term consumption. In order to reduce detection cost, a sensor based on the frequency resonance of a complementary split-ring resonator (CSRR) for crack detection has been presented in [11], but the contact measurement process reduced its efficiency. To make the probe light and maneuverable, an open-ended substrate-integrated waveguide probe has been proposed in [12]. The probe was based on amplitude testing, and its depth-detection capability was 3–6 mm. However, it cannot be widely used because of its narrow detection range. An array waveguide probe has been put forward for the orientation and sizing of surface cracks [13]. It can avoid large inspection times and unwanted noise, but it is costly compared to printed circuit board (PCB) technology. In addition, a sensor based on a ground plane defect has been presented in [14], which detected defects by monitoring resonance frequency shifts. The sensor was simple and highly sensitive, however, sweep-frequency detection made it more time-consuming. Otherwise, some defect detection systems have been improved by introducing artificial intelligence [15,16]. Undoubtedly, these methods were effective in solving some specific problems, but there is still some space to improve in cost, miniaturization and operational aspects.

Based on the available literature about microwave near-field testing methods in metallic surfaces, one can conclude the development trend. First, the microwave near-field sensors based on rectangular waveguides [7,17], substrate-integrated waveguides [12] and coaxial lines [18] have been implemented as amplitude detection probes that are simple, light and applicable to various conditions. Second, research has focused on loading artificially engineered electromagnetic materials (metamaterials) to improve the sensitivity and resolution of microwave near-field sensors [9,11]. Furthermore, research that utilized metamaterials for microwave near-field testing was mainly directed towards frequency shift detection but not for amplitude detection. In the meantime, the microstrip line sensor-loaded special resonance structure enormously improved the sensitivity by monitoring shifts in resonance frequencies [14]. However, research progress on reflection amplitude detection with high sensitivity developed slowly. In consideration of the advantages of handiness, easy integration and low cost, it is essential to design a highly sensitivity probe based on reflection amplitude detection [19]. 

Interdigital electrodes (IDEs) are mainly used for filter [20] and antenna [21] designs that are compact and light. Recently, IDE sensors have been used to monitor changes in dielectric materials [22] and gas sensors [23] and can also be used for the surface defect detection of metallic materials [24], due to the extraordinary feature of IDE sensors. The IDE sensors are sensitive enough to distinguish the variation of closed regions. It is particularly advantageous for defect detection, as it significantly reduces undesirable factors influencing sensor response.

Previously, there has been extensive use of the IDE structure for band-stop filters [20,25], and the microstrip-line-excited IDE exhibits band-stop characteristics. The minimum transmission coefficient frequency depends on the resonance frequency of the IDE. In order to prove that the IDE filter has the ability to detect surface defects in metals, an IDE microstrip band-stop filter, which was perpendicular to an aluminum plate with notch-type damage, was designed by the finite element method. The resulting shift in the minimum transmission frequency demonstrated that the IDE filter can be used for detecting defects. Therefore, when a plate surface is placed close to an IDE, various dimensions of damage disturb the electromagnetic field, resulting in various shifts in resonance frequency. Based on above facts, the IDE structure gives us discernment into designing sensitive surface probes for defect detection in metals.

This paper presents a novel sensor for notch-type damage detection based on the reflection amplitude in optimum frequency of the IDE structure. Essentially, the IDE probe works as a near-field sensor. It operates from 7.0 GHz to 14.5 GHz for notch-type damage detection in metals, including orientation and dimension detection. In addition, the depth and angle of the notch-type damage was detected through significant changes of the reflection coefficient, respectively. The IDE probe has a simple structure, is easy to operate, is multifunctional, and has a low fabrication cost.

## 2. Probe Design

**Figure 1:**
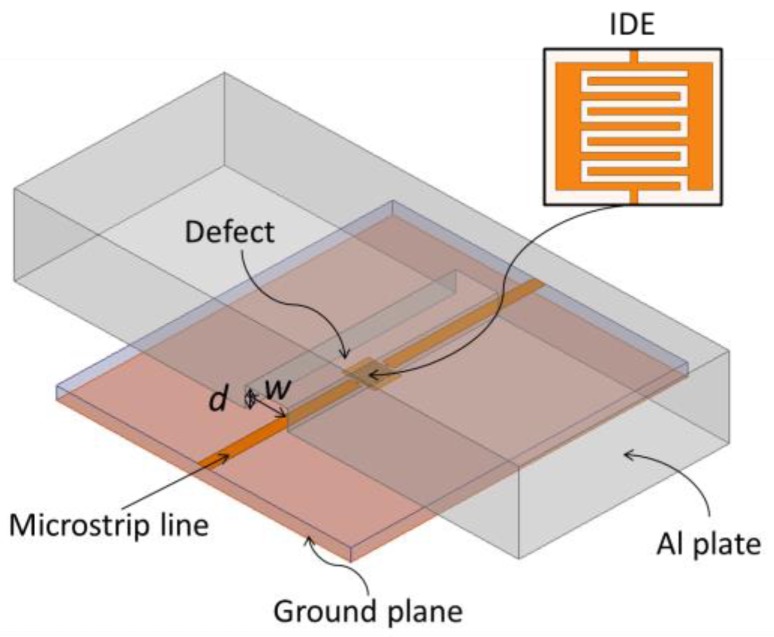
Schematic drawing of the microstrip IDE sensor monitoring an aluminum plate with surface notch-type damage.

In order to prove the IDE filter can detect surface damage in metals, we designed an IDE microstrip band-stop filter that is perpendicular to an aluminum plate with notch-type damage in ANSYS HFSS software, as shown in Figure 1. A thin Teflon film with a thickness of 0.05 mm is laid on the aluminum surface before the defect under film being scanned, where *w* and *d* are the width and depth of the defect, respectively. The filter adopts a rectangular IDE structure in the middle of the microstrip line, which makes it operate around 6 GHz. To achieve this goal, one can use the approximate model described in [26].

**Figure 2:**
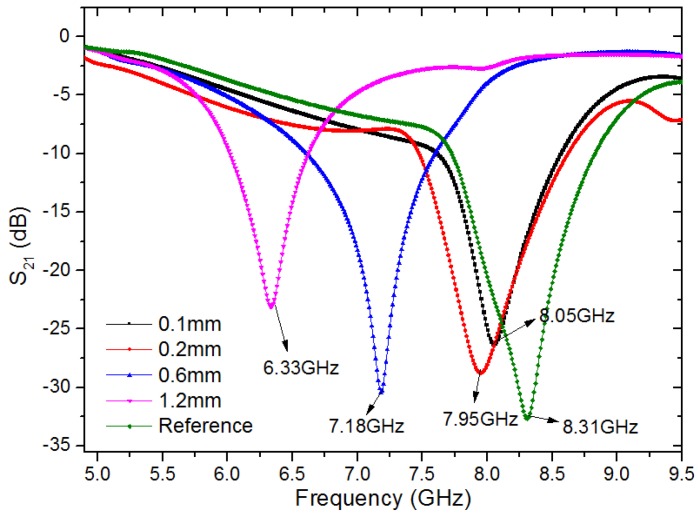
Scattering parameter of the IDE filter from full-wave simulation when encountering five different situations: aluminum blocks without a defect and with defects of 0.1 mm, 0.2 mm, 0.6 mm, and 1.2 mm in width, and 2 mm in depth for all cases.

When the sensor detects a plate with or without defects, the frequency of minimum transmission changes as shown in Figure 2. According to Figure 2, 6.33 GHz, 7.18 GHz, 7.95 GHz and 8.05 GHz are the resonance frequencies when the width of the notch-type damage is 1.2 mm, 0.6 mm, 0.2 mm, and 0.1 mm, respectively, and 8.31 GHz is the resonance frequency of the sensor when the surface is perfect. When the sensor passed over the notch-type damage with a width and depth of 0.1 mm and 2 mm, a shift of more than 260 MHz was observed with respect to the case without the defect, while a band-stop filter based on a complementary split-ring (CSRR) resonator with a shift of 210 MHz was observed in the same case. The notch-type damage was 0.2 mm in width and 2 mm in depth, and the sensor in this paper gave a resonance frequency shift of 360 MHz, which is more obvious than the resonance frequency shift realized by the CSRR sensor of 275 MHz [14]. The obvious shift in the minimum transmission frequency makes it a strong candidate for the detection of submillimeter-size defects in metallic surfaces. Based on the above analysis, the IDE structure is ideal for the design of sensitive surface probes. However, the IDE filter can only detect regions near the IDE structure, and the results will be disturbed when the defect is close to the micro-strip line. Thus, the micro-strip IDE filter is not adaptable as a scanning probe.

**Figure 3:**
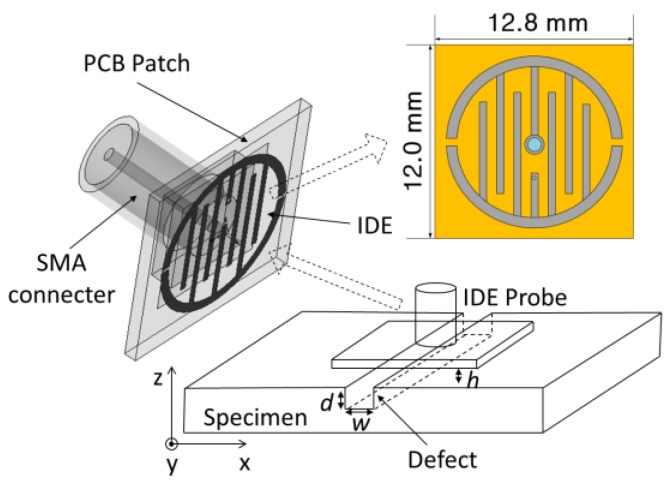
Schematic drawing of the IDE probe interrogating a metallic plate with long notch-type damage.

In this study, a single IDE structure was etched on an RC4003 substrate to avoid an over-complicated structure and undesirable interference. In order to detect the angle of the defect, the classic rectangular IDE is transformed into a round structure that makes the probe more compact. The IDE probe shown in Figure 3 has a cross section of 12 mm × 12.8 mm. A Sub-Miniature-A (SMA) connector is soldered on the back, and the seven interdigital strip lines of rounded contour are etched on the other side. The IDE probe is fed with 50 Ω coaxial line using the via-technology.

**Figure 4:**
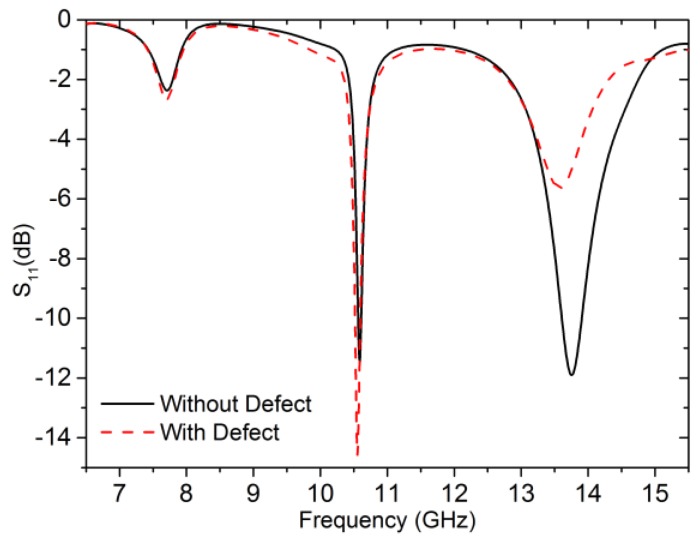
Reflection coefficients of the IDE probe with and without defect. The width and depth of the notch-type damage are both 4 mm. The standoff distance between the probe and the aluminum surface is 1mm for both cases.

**Figure 5:**
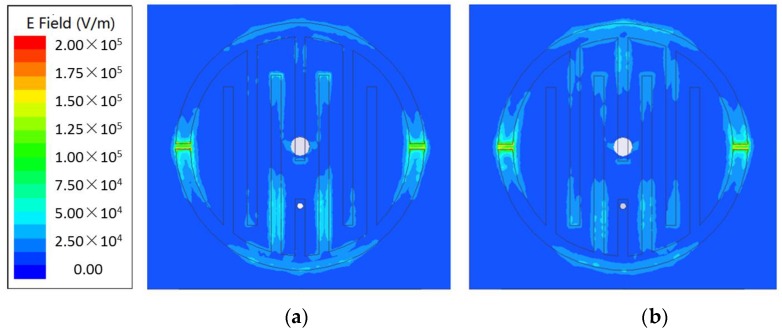
Electric field distributions on the surface of the PCB patch for different metallic surfaces, where the working frequency is 13.78 GHz. (**a**) Aluminum surface with defect; (**b**) Aluminum surface without defect.

In order to illustrate the behavior of the IDE structure and predict its resonance frequency, the simulation model setup in HFSS is shown in Figure 3. The SMA connector is included in electromagnetic simulation for more accurate results. Figure 4 presents the simulation results of reflection coefficients w**i**th and without defect in the plate surface within 6.5–15.5 GHz. A significant difference at around 13.78 GHz can be found. Figure 5 shows the electric field distribution on the IDE surface where the working frequency is 13.78 GHz. There is a noticeable difference between the aluminum board with a defect and that without a defect in the intermediate area of the electric field distribution. Keeping in view the above results, we may safely draw the conclusion that the defect can perturb the electromagnetic field around the IDE probe.

## 3. Detection Theory

Researchers have detected surface defects in metals using the microwave near-field method by the detection principle through the standing-wave shift [11] or the reflection coefficient change [13]. The IDE probe detects defects by monitoring the reflection coefficient change to reduce the detection time. Therefore, in order to explore the effect of defects in metallic surfaces on the behavior of the IDE probe, we considered this detection system as a capacitance model to explain the optimal frequency. When the specimen is regarded as a capacitor, it has complex relative permittivity. In this case, the reflection coefficient at the end of the sensor is expressed as

(1) $$\Gamma =\frac{1-j\omega Z_{0}\times C_{\left(\varepsilon_{s}\right)}}{1+j\omega Z_{0}\times C_{\left(\varepsilon_{s}\right)}}$$

where *Z*_0_ is the characteristic impedance of the sensor and *C*(*ε_s_*) is the capacitance of complex relative permittivity of the specimen, and ω is the angular frequency.

**Figure 6:**
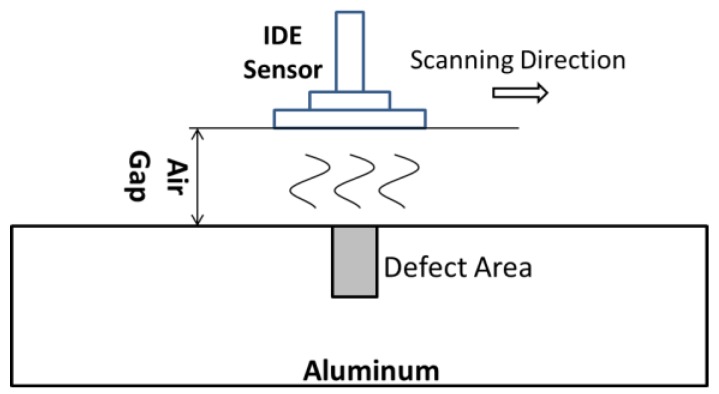
Configuration of specimen with surface defect, air gap and IDE sensor.

From Equation (1), the reflection coefficient is determined according to combinations of the sensor, the specimen, and the measuring frequency. If the metallic specimen surface has a defect under the sensor, this defect becomes another capacitor (as shown in Figure 6). Then, Equation (1) becomes more complicated:

$$\Gamma =\frac{1-j\omega Z_{0}\left[C_{\left(\varepsilon_{s}\right)}+C_{c}\right]}{1+j\omega Z_{0}\left[C_{\left(\varepsilon_{s}\right)}+C_{c}\right]}$$

where *C_c_* is the capacitance of the defect.

In this model, it has a standoff distance (*h*), and this air gap could change the reflection coefficient that is attributed to the system error. Therefore, when we use the microwave near field method to detect surface defects in metals, the standoff distance should be stationary [9,16].

## 4. Measurements Procedure

**Figure 7:**
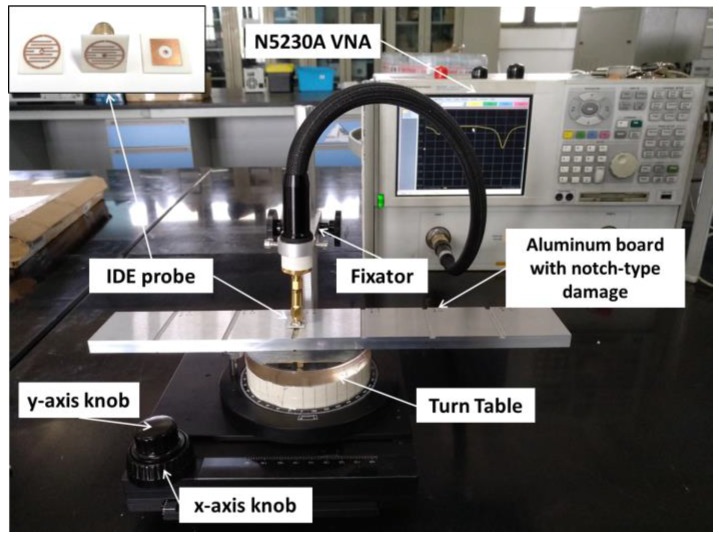
Photograph of the experimental configuration. The IDE probe is fixed on the fixator and the aluminum plate can be moved along the x-axis by turning the knob. The precision is 0.1 mm/step.

**Figure 8:**
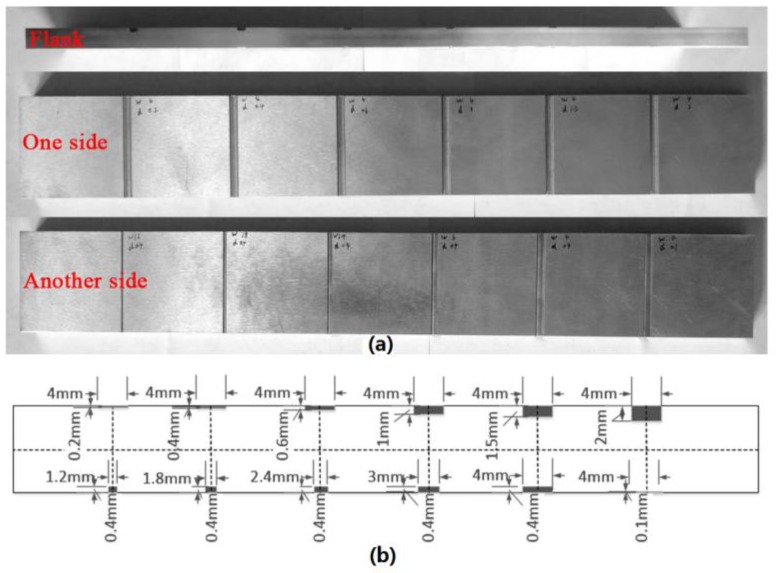
Schematic showing the aluminum sample. (**a**) Two sides of the aluminum plate; (**b**) Structure diagram of the aluminum plate.

The experimental setup is shown in Figure 7. The IDE probe is fixed on the fixator which can be used to adjust the standoff distance; the air gap is finally fixed at 1 mm. The specimen is put on the *x*–*y* stage. The *x*-axis knob is used to move the specimen along the probe, while the turntable is employed to rotate to a particular angle. In this work, the N5230A vector network analyzer (VNA) is used to record the magnitude of reflection coefficient. The IDE probe attached to an SMA connector is connected to the VNA. Figure 8 shows the artificial defects of the investigated specimen. The depth of defects increases with the same width on one side of the specimen, and the width of defects increases with the same depth (except the last one) on the other side. Before notch-type damage scanning (as shown in Figure 7), the IDE probe is located at a distance of 3 mm or further away from the defect, and the relative location of the probe is recorded. Then, the knob is rotated to move the plate along the probe, and the results of VNA for each step are recorded. A level instrument was used to ensure that their horizontal relative positions remain constant. When the plate moved away from the probe by about 3 mm, we finished the detection of this defect. Other defects are measured in the same way. When notch-type damage is encountered, a significant change in the reflection coefficient curve can be registered.

## 5. Result and Discussion

### 5.1. Scanning Detection

**Figure 9:**
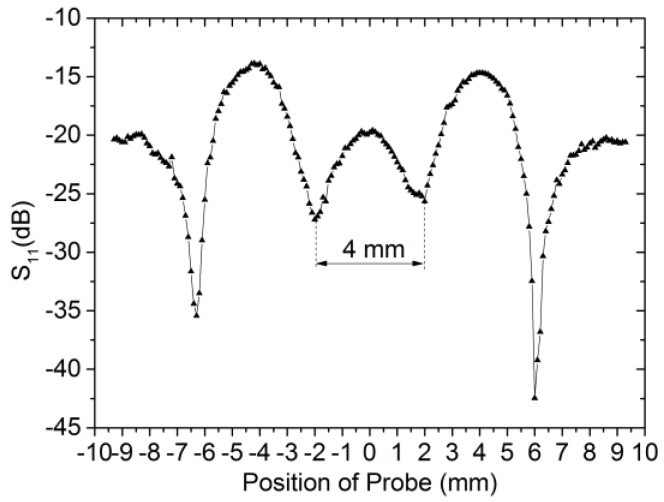
Experimental results of scanning the notch-type damage (*w* = 4 mm and *d* = 0.4 mm). The operation frequency is 13.86 GHz.

Figure 9 shows the experimental results of scanning the aluminum plate with long notch-type damage. The width and depth are 4 mm and 0.4 mm respectively, and the defect locates at 0 mm. According to Figure 9, there is a symmetry change in magnitude relative to the position of the notch-type damage at 13.86 GHz when the defect is encountered. The distance between the two troughs in the middle is close to the real value of the width.

In order to verify the capability of the IDE probe in width detection, other four defects in different width are scanned. The depth of the defects is 0.4 mm for all cases. The measured values are got by computing the distance between the two troughs in the middle. The actual width and measured value are shown in Table 1. The measured results are very close to the true value when the width is larger than 1.8 mm. However, the probe predicted width is 0.8 mm, while the actual width is 1.2 mm.

### 5.2. Orientation and Depth Detection

**Figure 10:**
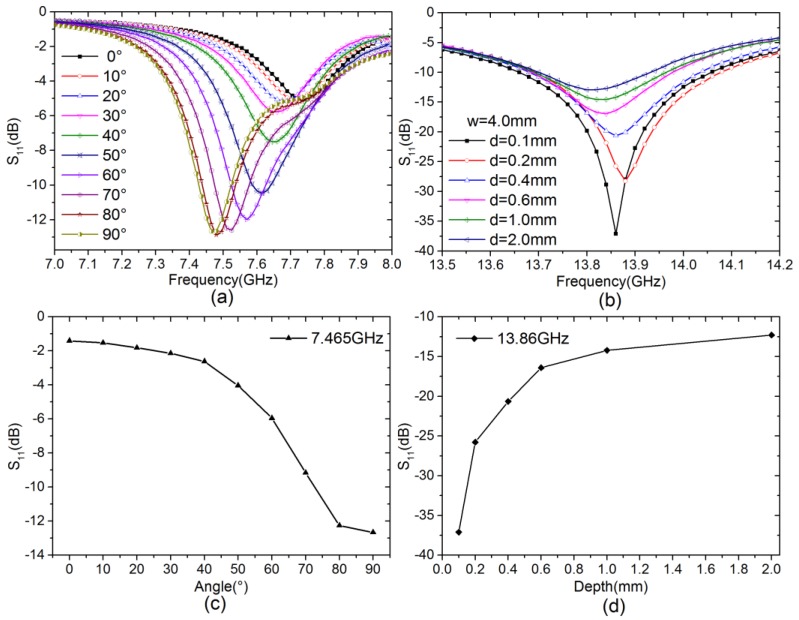
Experimental results for different condition. (**a**) Different angles (*w* = 4 mm and *d* = 2 mm); (**b**) Various depths (*w* = 4 mm); (**c**) Different angles and its amplitude at 7.465 GHz; (**d**) Various depths and its amplitude at 13.86 GHz.

To confirm the results associated with the orientation of notch-type damage, various angles (0°–90°) are detected. Referring to Figure 3, the defect was placed at the center of the probe’s interrogation aperture, and the *w* and *d* were set to 4 mm and 2 mm, respectively. The experimental results of various angles corresponding to various S-parameters are shown in Figure 10a, which shows that the probe output signals are indeed affected by orientation.

Prior to depth detects, its orientation can be done by rotating the plate until the amplitude of S_11_ attain the values corresponding to 0°. Various depths (0.1–2 mm) have been detected as shown in Figure 10b, while the width is 4 mm for all cases. Probe output signals are affected by depth, and various amplitudes of S-parameters corresponded to various depths.

From the results in Figure 10a, each angle (0–90°) corresponded to the unique amplitudes of the reflection coefficient. The magnitude decreases while angle increases within 7.2–7.47 GHz. As shown in Figure 10b, different depths correspond to a unique amplitude of the reflection coefficient; the magnitude decreases while depth decreases within 13.7–13.86 GHz. By adopting such simple monotonic relations, the orientation and depth value can be predicted by monitoring the magnitude of S-parameters at one frequency point. In order to select the frequency in which the amplitude change is monotonous and significant, we designed an optimization program in MATLAB based on the largest variances algorithm. In this program, we first pick out the frequency in which amplitude change is monotonous. Then, the variance coefficient for each frequency point is calculated and recorded. Finally, the biggest variance coefficient frequency was determined for detection. This is demonstrated by the experimental results shown in Figure 10c,d, and their estimated values show relative errors are less than 8% and 10% for angle and depth detection, respectively.

## 6. Conclusions

In this paper, a novel microwave IDE probe for detection, orientation and sizing of surface notch-type damage in metallic surfaces has been outlined. The probe utilizes IDE technology to make the probe compact and sensitive. The detection results indicated that scanning detection based on an IDE probe can be used to predict the width when the width is larger than 1.8 mm. In addition, the angle and depth of the defect can be detected by recording the change in magnitude at a single frequency point. Compared to the other microwave methods presented in literature, the IDE probe is multifunctional, relatively inexpensive and sensitive in amplitude detection. To summarize, the microwave IDE probe is promising for the nondestructive evaluation of metallic structure surfaces, and for other applications such as material detection and characterization.

## Figures and Tables

**Table 1:** Comparison of actual width and measured value.

Actual Width (mm)	Measured Value (mm)	Relative Error
3.0	3.2	6.7%
2.4	2.6	8.3%
1.8	2.0	11.1%
1.2	0.8	33.3%
